# A Possible Mechanism of Zika Virus Associated Microcephaly: Imperative Role of Retinoic Acid Response Element (RARE) Consensus Sequence Repeats in the Viral Genome

**DOI:** 10.3389/fnhum.2016.00403

**Published:** 2016-08-09

**Authors:** Ashutosh Kumar, Himanshu N. Singh, Vikas Pareek, Khursheed Raza, Subrahamanyam Dantham, Pavan Kumar, Sankat Mochan, Muneeb A. Faiq

**Affiliations:** ^1^Department of Anatomy, All India Institute of Medical SciencesNew Delhi, India; ^2^Department of Biochemistry, All India Institute of Medical SciencesNew Delhi, India; ^3^Computational Neuroscience and Neuroimaging Division, National Brain Research CentreManesar, India; ^4^Dr. Rajendra Prasad Centre for Ophthalmic Sciences, All India Institute of Medical SciencesNew Delhi, India; ^5^Laboratory for Molecular Reproduction and Genetics, Department of Anatomy, All India Institute of medical SciencesNew Delhi, India; ^6^Medical Biotechnology Laboratory, Dr. B. R. Ambedkar Centre for Biomedical Research, University of DelhiNew Delhi, India

**Keywords:** fetal brain malformation, microcephaly, neurotropism, retinoic acid response element (RARE), zika virus (ZIKV)

## Abstract

Owing to the reports of microcephaly as a consistent outcome in the fetuses of pregnant women infected with ZIKV in Brazil, Zika virus (ZIKV)—microcephaly etiomechanistic relationship has recently been implicated. Researchers, however, are still struggling to establish an embryological basis for this interesting causal handcuff. The present study reveals robust evidence in favor of a plausible ZIKV-microcephaly cause-effect liaison. The rationale is based on: (1) sequence homology between ZIKV genome and the response element of an early neural tube developmental marker “retinoic acid” in human DNA and (2) comprehensive similarities between the details of brain defects in ZIKV-microcephaly and retinoic acid embryopathy. Retinoic acid is considered as the earliest factor for regulating anteroposterior axis of neural tube and positioning of structures in developing brain through retinoic acid response elements (RARE) consensus sequence (5′–AGGTCA–3′) in promoter regions of retinoic acid-dependent genes. We screened genomic sequences of already reported virulent ZIKV strains (including those linked to microcephaly) and other viruses available in National Institute of Health genetic sequence database (GenBank) for the RARE consensus repeats and obtained results strongly bolstering our hypothesis that ZIKV strains associated with microcephaly may act through precipitation of dysregulation in retinoic acid-dependent genes by introducing extra stretches of RARE consensus sequence repeats in the genome of developing brain cells. Additional support to our hypothesis comes from our findings that screening of other viruses for RARE consensus sequence repeats is positive only for those known to display neurotropism and cause fetal brain defects (for which maternal-fetal transmission during developing stage may be required). The numbers of RARE sequence repeats appeared to match with the virulence of screened positive viruses. Although, bioinformatic evidence and embryological features are in favor of our hypothesis, additional studies including animal models are warranted to validate our proposition. Such studies are likely to unfold ZIKV-microcephaly association and may help in devising methods to combat it.

## Introduction

ZIKV has recently been a hot topic among researchers as well as general public due to its extensive geographical distribution and perceived health related threats; though most of the cases, as of now, are being reported from Brazil (Cipriano and Monteiro, 2016; Samarasekera and Triunfol, 2016). One of the main health intimidations by ZIKV has been microcephaly in the fetuses born of the infected women. There is division of opinion among researchers regarding ZIKV-microcephaly association (Rasmussen et al., 2016). This is despite the fact that ZIKV has been detected in the placenta and amniotic fluid of fetuses born with microcephaly (Calvet et al., 2016; Mlakar et al., 2016; Schuler-Faccini et al., 2016). It has also been detected in the brain of a fetus died of severe brain defects. There is strong epidemiological evidence of ZIKV–microcephaly association (Schuler-Faccini et al., 2016; WHO | Zika situation report, 2016). It would, as is obvious, be hard to justify that a true cause-effect relationship exists between ZIKV and microcephaly until its neuro-embryological basis is established. The present study has made an attempt to identify and explain a plausible embryological basis of ZIKV-microcephaly association. We chose retinoic acid for exploring its involvement in ZIKV–microcephaly relationship because of the wide-ranging similarities between brain malformations caused by retinoic acid signaling dysregulation and the brain defects observed in ZIKV infected fetuses (as provided in many important reports; Aragao et al., 2016; Calvet et al., 2016; Hazin et al., 2016; Mlakar et al., 2016).

### Retinoic acid-mediated mechanism in neural tube formation and further brain development

Retinoic acid is a non-peptide small lipophilic molecule derived from retinol—an active ingredient of vitamin A. Retinol gets converted to retinal and further into retinoic acid by the action of dehydrogenases which includes CYP1B1. Retinoic acid is taken to the nucleus by the cellular retinoic acid binding protein (CRABP) where it binds to retinoic acid receptors (RAR and RXR) on the promoter regions of specific genes (Balmer and Blomhoff, 2002; Rhinn and Dollé, 2012). RAR and RXR are important transcription factors (Rhinn and Dollé, 2012) and actively influence RARE consensus sequences in promoter regions of a plethora of genes involved in neural tube development in addition to serving other pertinent embryological functions (Balmer and Blomhoff, 2002; Rhinn and Dollé, 2012). These genes further activate a cascade of regulatory molecules involved in neural tube formation and brain development. Any dysregulation of this intricate molecular process at any step can lead to various degrees of neural tube defects and brain malformations (Grapin-Botton et al., 1998; Kam et al., 2012; Rhinn and Dollé, 2012). The RARE sequence is also spread all over human genome through commonly found ALU repeats which contain RARE sequence (Vansant and Reynolds, 1995). Retinoic acid enjoys the stature of being the initial-most molecular factor involved in the determination of the neural axis and, is the prime determinant of the anteroposterior axis owing to its interplay with Nodal gene in developing neural tube (Durston et al., 1989; Kam et al., 2012). Together with fibroblast growth factor (FGF), it is known to set a code for establishing the anterio-posterior axis (instituted by opposing gradients of retinoic acid and FGF along the neural tube) which is followed by other molecular factors (Martínez-Morales et al., 2011; Shimozono et al., 2013).

Nodal, which is thought to be the master regulator gene for deciding the axes of the developing neural tube has RARE consensus sequence in its intron-1. Retinoic acid is also involved in deciding dorsoventral axis by intricate crosstalk with Nodal (Kam et al., 2012). A plethora of literature suggests that retinoic acid is involved in FGF8 repression during body axis extension and regulation of the homeobox (HOX) genes involved in determining the position of the neural segments (Grapin-Botton et al., 1998). More than deciding the axes and positioning of the neural segments at the time of neural tube formation, retinoic acid (at a later stage) also has imperative role in the development of midline, posterio-dorsal brain structures, and forebrain components in addition to corticogenesis. Disruptions in the retinoic acid signaling pathway have been implicated in ensuing brain defects in developing fetus involving all the above mentioned structures and may, arguably, present as microcephaly (Wilson et al., 2007; Siegenthaler et al., 2009; Crandall et al., 2011; Gupta and Sen, 2015). Retinoic acid is also a teratogen and can cause the neural tube defect and other brain malformations if provided in abnormal doses to developing fetus (Yamamoto et al., 2003).

### Plausible mechanism of ZIKV mediated retinoic acid signaling dysregulation resulting in fetal microcephaly

**Figure 1:**
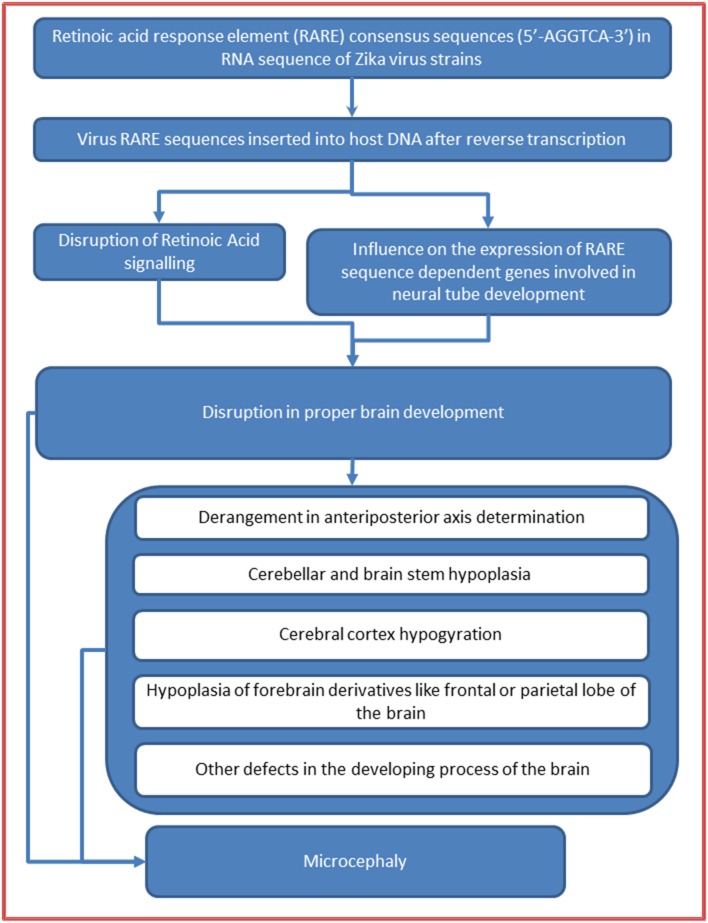
**Retinoic acid response element (RARE) mediated mechanism in Zika virus microcephaly association**.

ZIKV is a positive sense RNA Flavivirus (Baronti et al., 2014). When an RNA virus infects a host cell, its genetic material is reverse-transcribed into cDNA by reverse transcriptase which, in turn, synthesizes complementary sense DNA strand, and finally, the so composed DNA gets integrated into the host DNA. We proceed with an important and coherent line of arguments that in acute viremia heavy doses of viral DNA (reverse-transcribed from RNA) will be integrating into the host DNA, and if the inserted DNA sequences match the regulatory regions of certain host genes then they may influence their expressions. By the transitive property of similarities, it can then be justified that, the RARE consensus sequence repeats (if present) in the genomic sequences of ZIKV strains would be inserted into the promoter regions of RARE dependent genes of the host DNA and, therefore, may influence their expression in a way that the developing fetus manifests with brain malformation like microcephaly (Figure 1).

Current understanding of the ZIKV-microcephaly association is improving as more and more relevant reports are coming up and many recent studies strongly agree to the notion that ZIKV-microcephaly may be a developmental brain malformation rather than a mere brain destruction by the virus (Hazin et al., 2016; Mlakar et al., 2016). So, in the light of comprehensive similarities between fetal brain defects in ZIKV infection and brain malformations caused by retinoic acid dysregulation in developing fetus, we considered it a plausible rationale to search for a possible retinoic acid-mediated mechanism involved in ZIKV-microcephaly association. We, for this important reason, searched for the RARE consensus sequence (5′–AGGTCA–3′) repeats in the genomic sequences of the ZIKV strains with a hypothesis that the virus might act through disruption of normal retinoic acid signaling mediated by incorporation of these sequences into the DNA of developing host brain cells. To validate this hypothesis robustly and to establish the cogency of our study, we also screened some other viruses (including members of the flaviviridae family and those viruses which are known to have maternal-fetal/perinatal transmission either causing congenital brain malformations or showing neurotropism) and subjected them to the same analysis.

## Materials and methods

The genome sequence information of 17 strains of ZIKV (including “Brazil ZKIV-2015” which was recently implicated in causing microcephaly in Brazilian fetuses; Calvet et al., 2016; Mlakar et al., 2016), and many other viruses were retrieved from the NCBI/GenBank database (http://www.ncbi.nlm.nih.gov/genbank/) (Table 1) and screened for the presence of RARE consensus sequence (5′–AGGTCA–3′). Among the viruses other than ZIKV, selection was made under four groups viz: (1) Members of the flaviviridae family other than ZIKV (to check if RARE consensus sequence repeats were essentially present in the family) (2) Viruses showing maternal-fetal/perinatal transmission and causing congenital brain malformations (3) Viruses showing neurotropism but not known to cause fetal brain defects (4) Viruses chosen randomly from the RNA/DNA viruses not fitting in any of the above three groups (to account for the selection bias and to establish coherence and validity of the screening process). To identify the enrichment of RARE sequence (5′–AGGTCA–3′) repeats present in the viruses, a Perl script was written and executed.

**Table 1:** **The ZIKV and other virus strains included in the study**.

**Flaviviridae Family (With GenBank accession numbers)**		**Other than Flaviviridae family viruses (with GenBank accession numbers)**		
**Zika virus strains**	**Other Flaviviridae members**	**Show maternal-fetal/perinatal transmission and cause congenital brain malformations**	**Show neurotropism but not known to cause fetal brain defect**	**RNA/DNA viruses not to be fit in the other three groups**
**KU497555** (Brazil ZIKV-2015)	Dengue virus (Type I–IV)	Human cytomegalovirus (Human herpesvirus 5)	Measles virus (KJ410048.1)	Hepatitis Delta virus (NC_001653.2)
	EF025110.1			
	AF489932.1			
	AB214882.1			
	AY762085.1			
AY632535	Chikungunya virus^**^ (JX088705.1)			
DQ859059	Japanese Encephalitis virus (AY303791.1)	HIV Type 1 (NC_001802.1)	Mumps virus (KM597072.1)	Hepatitis B virus (AB937799.1)
EU545988^*^	West Nile virus (M12294.2)	Rubella virus (NC_001545)	Human poliovirus 2 (KJ419277.1)	Hepatitis A virus (AB279735.1)
NC_002031.1	Yellow fever virus (NC_002031.1)	Herpes simplex virus type 1 (NC_001806.2)	Ebola virus NC_001806.2	Human rhinovirus 14 NC_001490.1
HQ234500	Estern Equine encephalitis Virus (KJ659366.1)	Herpes simplex virus type 2 (strain HG52) (Z86099.2)	Rabies virus (EF206718.1)	Human Papilloma virus type 1a HPU06714
HQ234501		Herpes simplex virus type 3 (Varicella Zoster) (KC112914.1)	Yellow fever virus (NC_002031.1)	
JN860885^*^			Human Corona virus HKU1 (KF430201.1)	
KF268948				
KF268949				
KF268950				
KF993678				
KJ634273				
KJ776791^*^				
KU312315				
LC002520				
Z1106033				

* Share close genomic sequence homology with **KU497555** strain (Brazil ZIKV-2015);

** *Maternal-fetal transmission in human reported*.

**Figure 2:**
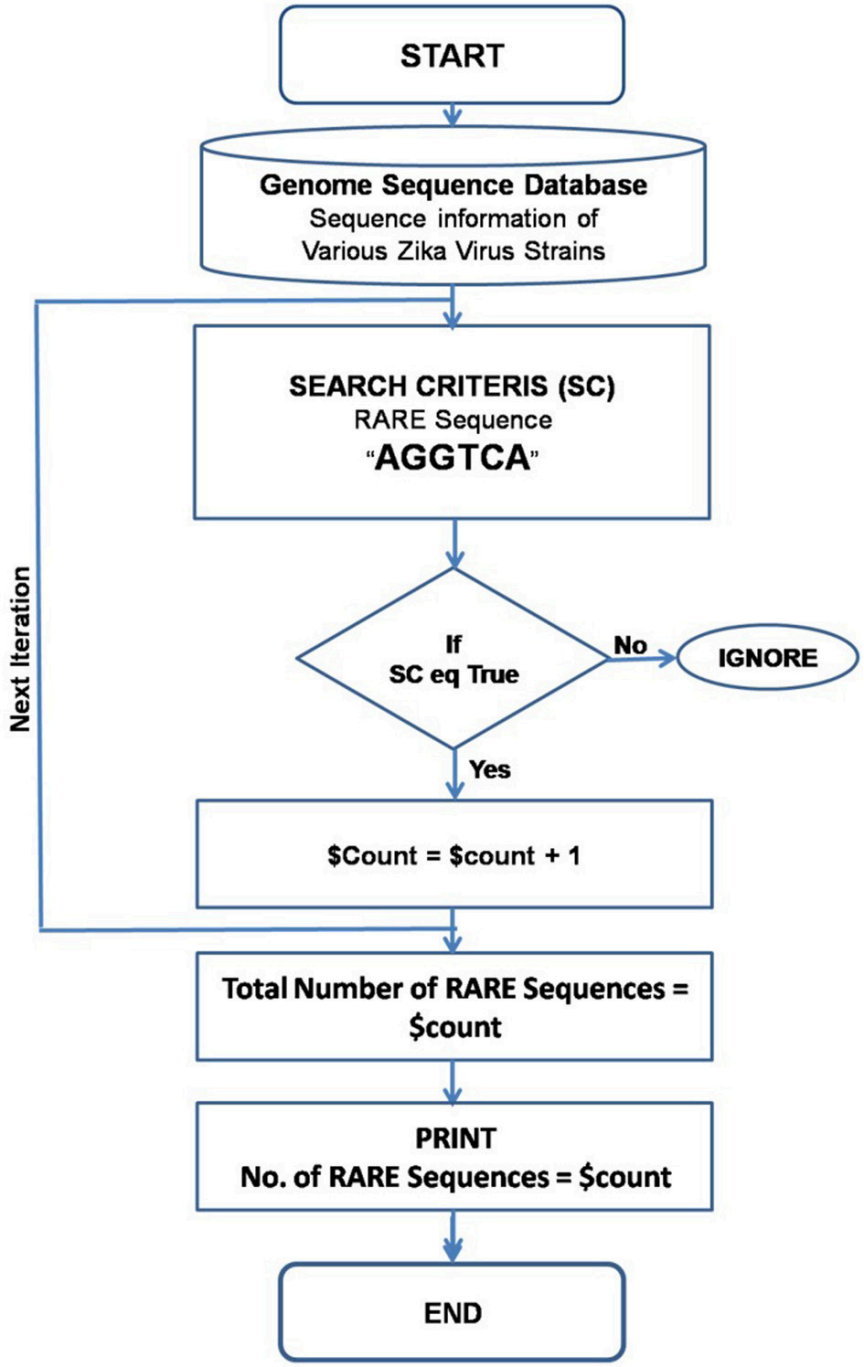
**Flow chart for the execution of the PERL script written to search and identify the consensus RARE sequences**.

In order to find and count the RARE sequences from the ZIKA virus genome, a PERL script was generated entitled “*RARE_Seq_finder.pl*.” The PERL script was based on the window shift algorithm. Genome sequence information of ZIKA virus isolates was downloaded from various genome databases. As input, the PERL Script requires only the list file carrying names of the genome sequence files. It searches the RARE sequences globally in the genome, and lists all genome file names along with number of RARE sequences present in the genome. A complete flow-chart with all the steps involved in the counting the RARE sequences is shown in Figure 2. The complete PERL script for this study is available as Supplementary Material.

## Result

The ZIKV strains associated with microcephaly contained RARE consensus sequence repeats. The number of repeats corresponds to the virulence of each strain i.e., greater the number of RARE repeats higher the chances of microcephaly. Complete details of the number of RARE sequence repeats in each ZIKV strain are given in Table 2. For viruses other than ZIKV, the RARE consensus sequence repeats were found to be present only in those viruses known for maternal-fetal/perinatal transmission, involved in fetal brain defects and displaying neurotropism. The numbers of RARE sequence repeats was directly proportional to the virulence of screened viruses. The number of RARE consensus sequences repeats in the checked 17 strains of the ZIKV and other ZIKV viruses are provided in Tables 2, 3 respectively.

**Table 2:** **Number of RARE consensus sequence (5′–AGGTCA–3′) repeats in genomic sequences of ZIKV strains**.

	**ZIKV Strains**	**Number of RARE Sequence (5′–AGGTCA–3′) repeats**
I	**KU497555** (Brazil ZIKV-2015)	**4**
Ii	AY632535	2
Iii	DQ859059	3
Iv	EU545988	4
V	HQ234499	3
Vi	HQ234500	2
Vii	HQ234501	3
Viii	JN860885	4
Ix	KF268948	3
X	KF268949	2
Xi	KF268950	3
Xii	KF993678	4
Xiii	KJ634273	0
Xiv	KJ776791	4
Xv	KU312315	0
Xvi	LC002520	2
Xvii	Z1106033	4

**Table 3:** **Number of RARE consensus sequence (5′–AGGTCA–3′) repeats in genomic sequence of other viruses**.

**Other Viruses Strains (GenBank accession number in brackets)**	**Type**	**Length of reading frame**	**Number of RARE Sequences (5′–AGGTCA–3′)**	**Neurotropism**	**Congenital Brain Defect**	**References**
Dengue virus (Type I–IV)	(+)ssRNA, linear			+	−	Tuiskunen et al., 2011; Salazar et al., 2013
(EF025110.1)		10735 bp	Type 1–3			
(AF489932.1)		10722 bp	Type 2–0			
(AB214882.1)		10707 bp	Type 3–2			
(AY762085.1)		10649 bp	Type 4–6			
^*^Chikungunya virus (JX088705.1)	(+)ssRNA, linear	11811 bp	4	+	+	Koyuncu et al., 2013; Gérardin et al., 2014
Japanese Encephalitis virus (AY303791.1)	(+)ssRNA, linear	10970 bp	6	+	−	Kimura-Kuroda et al., 1993
West Nile virus (M12294.2)	(+)ssRNA, linear	10962 bp	2	+	−	Samuel et al., 2007
Rabies virus (EF206718.1)	(+)ssRNA, linear	11933 bp	3	+	−	Tsiang et al., 1983
Yellow fever virus (NC_002031.1)	(+)ssRNA, linear	10862 bp	3	+	−	Findlay and Stern, 1935; Guimard et al., 2009
Estern Equine encephalitis Virus (KJ659366.1)	(+)ssRNA, linear	11613 bp	5	+	−	Ludlow et al., 2016
^*^Human cytomegalovirus (Human herpesvirus 5) (NC_006273.2)	ds-DNA, linear	235646 bp	40	+	+	Teissier et al., 2014
^*^HIV Type 1 (NC_001802.1)	(+)ssRNA, linear	9181 bp	2	+	+	Civitello, 1991; Koyuncu et al., 2013
HIV Type 2 (NC_001722.1)	(+)ssRNA, linear	10359 bp	0	^**^	−	Wood et al., 2012
^*^Rubella virus (NC_001545)	(+)ssRNA, linear	9762 bp	3	+	+	Koyuncu et al., 2013; Yazigi et al., 2016
Mumps virus (KM597072.1)	(−)ssRNA, linear	15263 bp	4	+	−	Takano et al., 1999; Rubin et al., 2003
^*^Herpes simplex virus type 3 (Varicella Zoster) (KC112914.1)	dsDNA, linear	124884 bp	8	+	+	Scheffer et al., 1991; Sauerbrei and Wutzler, 2000
Herpes simplex virus type 1 (NC_001806.2)	dsDNA, linear	148709 bp	26	+	+	Straface et al., 2012; Koyuncu et al., 2013
^*^Herpes simplex virus type 2 (strain HG52) (Z86099.2)	ds-DNA, linear	154746 bp	20	+	+	Straface et al., 2012; Koyuncu et al., 2013
Hepatitis C virus (subtype 1 g) AM910652	(+)ssRNA, linear	9490 bp	4	+	+	Forton et al., 2001; Grover et al., 2012; Koyuncu et al., 2013
Human Corona virus HKU1 KF430201.1	(+)ssRNA, linear	29934 bp	3	+	−	Lau et al., 2006
SARS Corona virus ExoN1 (FJ882956.1)	(+)ssRNA, linear	29644 bp	3	+	−	Xu et al., 2005
Ebola virus (KU182909.1)	(−)ssRNA, linear	18959 bp	5	+	−^***^	Mupapa et al., 1999; Koyuncu et al., 2013
Human Papilloma virus type 1a (HPU06714)	ss-RNA, circular	1682 bp	0	−	−	
Hepatitis E Virus (AB220977.1)	(+)ssRNA, linear	7266 bp	0	−	−	
Hepatitis B virus (AB937799.1)	ds-DNA, linear	3213 bp	0	−	−	
Hepatitis A virus (AB279735.1)	(+)ssRNA, linear	7478 bp	0	−	−	
Human rhinovirus 14 (NC_001490.1)	(+)ssRNA, linear	7212 bp	0	−	−	
Adult Rota virus (NC_007553.1)	(+)dsRNA, linear	1287 bp	0	−	−	

* Known to cause microcephaly;

** Rare cases of encephalitis noted;

*** *No data available due to early fetal loss and/or non-evaluation of the dead still birth baby for brain defects*.

## Discussion

The RARE consensus sequence repeats were found in most of the ZIKV strains but the frequency of the repeats varied among the strains. Surprisingly, the ZIKV strain (KU497555.1, Table 2) which was implicated in the recent cases of the microcephaly in Brazil (Calvet et al., 2016), and other strains which shared close homology with it contained the highest number of RARE sequence repeats (Table 2). The genomic sequence of the Brazilian ZIKV strain was found to share near 100% homology to the Polynesian strain (KJ776791, Table 2) which was taken as a reference in a recent study (Calvet et al., 2016). Another study (Mlakar et al., 2016) which had retrieved the virus from the brain tissue of a dead fetus with microcephaly had similar interpretation that the retrieved strain had a very close homology to the Polynesian strain (99.7%) and two other strains (EU545988-98%, JN860885-98.3%). All the three strains were amongst the highest number of RARE sequence containing strains as revealed in our study (Table 2). Although, it would still be a far-fetched conjecture to consider an association of these ZIKV strains (with the highest number of RARE sequence repeats) to microcephaly unless ZIKV-microcephaly link is well established.

From analysis of the data from all the viruses other than ZIKV (Table 3), it is clear that only those viruses contain the RARE consensus sequence repeats which are either known to cause fetal brain malformations or show neurotropism. Different strains of the same genus vary in the presence of these sequence repeats and it is absent altogether in some strains. A virus with comparatively higher RARE sequence repeats seems to be a more potent teratogen (in terms of causing congenital brain malformation) or displays correspondingly robust neurotropism as suggested by the analysis of Table 3. Some of these viruses are well known to cause fetal microcephaly also (Table 3, single star marked). Almost all of the viruses which contained RARE consensus sequence essentially show neurotropism of some degree (as documented in the literature) except the Dengue virus type-2 in which we found no RARE sequence repeats despite the evidence of its neurotropism in literature (Salazar et al., 2013). The presence of the RARE consensus sequence repeats revealed in the known neurotropic viruses (Table 3) could be an indication that retinoic acid signaling may have an important role to play in neurotropism, although we couldn't find sufficient support in literature for this claim due to paucity of research over this issue. There is, nevertheless, substantial evidence that retinoic acid inducible gene (RIG)-1 which is upregulated by retinoic acid, functions as an intra-cellular pattern recognition receptor for the known neurotrophic receptor viruses (Furr and Marriott, 2012; Carty et al., 2014). A recent study reports the involvement of a similar viral pattern recognition receptor TLR-3 in ZIKV caused growth restriction and depletion of neural stem cell progenitors in human cerebral organoids (Dang et al., 2016). There are additional reports suggesting a necessary RIG-1 and TLR-3 interaction to surge innate immunity in response of viral infections (Liu et al., 2007; Slater et al., 2010). These reports indicate the involvement of retinoic acid signaling in proper brain development. Further research is required to establish the nature of retinoic acid-neurotropism relationship but based on our observations on the ZIKV strains and various other viruses, it seems coherent to consider the presence of RARE consensus repeats as the reason for the obligatory neurotropic character of the ZIKV strains implicated in microcephaly (Calvet et al., 2016; Hazin et al., 2016; Mlakar et al., 2016).

The high frequency of RARE consensus repeats in some double stranded DNA viruses may be because of larger reading frame length and probably a RARE sequence index value (which balances number of repeats with total reading frame length of genomic sequence). This index could be an appropriate measure for representing potency of a virus for causing congenital brain malformation or neurotropism. A further detailed study on the presence of RARE consensus sequence repeats in ZIKV vs. other viruses may be desired to confirm if retinoic acid signaling dysregulation is a common mechanism used by such viruses to cause congenital brain malformations.

### Fetal brain defects in ZIKV-microcephaly closely resemble to brain malformation caused by dysregulation of retinoic acid signaling

Fetal microcephaly has always been known as a developmental anomaly caused by multiple etiologies (Mochida and Walsh, 2001). Retinoic acid signaling dysregulation has also been implicated as a common mechanism in many environmental or developmental reasons of microcephaly (Lammer et al., 1985; Kot-Leibovich and Fainsod, 2009; Paganelli et al., 2010). The anatomical details of the brain of the fetuses suspected of ZIKV infection in recent reports (Aragao et al., 2016; Calvet et al., 2016; Hazin et al., 2016; Mlakar et al., 2016) closely resemble to those caused by retinoic acid signaling dysregulation and have many features in common i.e., cerebellar and brain stem hypoplasia (Yamamoto et al., 2003), ventriculomegaly (Micucci et al., 2014), hypoplasia of forebrain derivatives like frontal or parietal lobe of the brain (Halilagic et al., 2007), and hypogyration of cerebral cortex (Siegenthaler et al., 2009). The reason behind these striking resemblances could be that the retinoic acid signaling is crucially involved in development of the dorsal brain structures (Wilson et al., 2007; including cerebellum and brain stem), midline forebrain structures (Gupta and Sen, 2015; including choroid plexus which synthesizes CSF and a malformation of it may result in hydrocephalus and in turn ventriculomegaly), and the cortex (Siegenthaler et al., 2009; Crandall et al., 2011). So it is an obviously plausible concept in the light of our hypothesis as to why retinoic acid signaling dysregulation may result in malformations similar to that noted in ZIKV caused microcephaly (Calvet et al., 2016; Hazin et al., 2016; Mlakar et al., 2016).

The presentation of the brain defects in retinoic acid dysregulation varies depending on the stage of embryonic development. If the signaling disruption ensues at late gastrulation or early neurulation stage, the defect should be occurring in rostrocaudal sequencing of the brain structures in the developing neural tube i.e., there may be a larger hindbrain at the expense of anterior brain structures. But if the disruption is at late developmental stages that will affect dorsalization of the brain or hindbrain structures i.e., may cause cerebellar or brain stem hypoplasia or absence of some hind brain components (Wilson et al., 2007).

The presence of microcephaly or other brain malformations in the fetuses born of the ZIKV infected mothers (similar to the other viruses causing congenital brain malformations) may also depend upon the time-point of the fetal infection during neural tube formation, and also on the total viral load (Adams Waldorf and McAdams, 2013). In some recent reports of the cases with ZIKV-microcephaly, pregnant women presented with clinical features of ZIKV infection at 10th week of gestation or beyond and microcephalic features were detected on ultrasound beyond 20th week (Calvet et al., 2016; Mlakar et al., 2016). This matches with the retinoic acid signaling dysregulation at the late developmental stages when dorsal brain structures and hindbrain brain structures are still in development and cortical gyration still in progress. A disruption at this stage would essentially result in the features typically seen in ZIKV microcephaly (Calvet et al., 2016; Hazin et al., 2016; Mlakar et al., 2016) as has been noted above.

Due to striking resemblance to the embryological malformations, the recent reports on ZIKV-microcephaly (Calvet et al., 2016; Hazin et al., 2016; Mlakar et al., 2016) have stressed upon considering the fetal brain damage as a developmental problem rather than the brain destruction caused by viral invasion of neuronal cells. One important study has found substantial evidence of presence of ZIKV in the neurons through immuno-histological and microbiology investigations of the fetal brain tissue died of the severe microcephaly (Mlakar et al., 2016). Interstingly, other probable infectious causes of the microcephaly were successfully ruled out in this study.

### Justification from maternal-fetal transmission

On an interesting note, our data also suggests that a virus which contains RARE consensus sequence repeats also shows some degree of neurotropism but its causal handcuff to microcephaly or any other type of fetal brain defect essentially depends upon its ability for maternal-fetal transmission. Chikungunya virus ((+)ssRNA, linear, 11811 bp: Table 3) seems to be a representative example. It contains four RARE consensus sequence repeats and shows neurotropism (Koyuncu et al., 2013), maternal-fetal transmission in humans has also been sufficiently demonstrated for this virus (Fritel et al., 2010; Gérardin et al., 2008), and is also known to cause microcephaly and/or other fetal brain defects (Gérardin et al., 2014). On the other hand Japanese encephalitis virus ((+)ssRNA, linear, 10970 bp: Table 3) which has no known evidence of maternal-fetal transmission in humans, although having shorter frame length and more number of RARE consensus sequence repeats (numbering to six) is known to show neurotropism (Kimura-Kuroda et al., 1993) but do not cause fetal brain defect in humans. The obligatory nature of ability of maternal-fetal transmission in a virus to cause fetal brain damage is empirically coherent and further suggests that presence of RARE consensus sequence repeats in a virus genome may be limited to confer it neurovirulence only, and may require additional contributing factors toward its maternal-fetal transmission ability.

### Further notes in support of our hypothesis concerning ZIKV-mediated microcephaly

Another evidence in favor of the retinoic acid signaling dysregulation as a probable mechanism in ZIKV- microcephaly is that the mouse model study of a well-known fetal anomaly syndrome called “CHARGE” (in which dysregulation of retinoic acid signaling has been implicated) reported some anomalous features very similar to that found in fetal brains suspected of ZIKV microcephaly (Calvet et al., 2016; Hazin et al., 2016; Mlakar et al., 2016) such as cerebellar hypoplasia and ventriculomegaly (Micucci et al., 2014). In addition to that, hydrocephalus and malformation of corpus callosum noted in some cases of ZIKV microcephaly (Aragao et al., 2016) are also present in “CHARGE” syndrome. Furthermore, the widespread cortical anomalies in ZIKV microcephaly get explained by an interesting study involving retinoic acid (Siegenthaler et al., 2009). These investigators reported cranial meninges as a source of retinoic acid to the developing cortex (radial glial cells carrying migrating progenitor cells to the upper cortical layers attach their end feet to the covering meninx, from where they get retinoic acid as a diffusible morphogen and transfer to other cells involved in corticogenesis). Retinoic acid signaling has been extensively implicated in cortical genesis (Harrison-Uy et al., 2013), especially in the migration of progenitor cells to the upper cortical layers riding on the radial glial cells (Siegenthaler et al., 2009; Crandall et al., 2011). Many contemporary reports have confirmed that ZIKV has detrimental effects on human cortical progenitor cells in neural stem cell cultures (Crandall et al., 2011) and neurospheres or brain organoids developed from human induced pluripotent cells (Garcez et al., 2016). ZIKV has also been shown to induce cell death and attenuate growth mediated through vast transcriptomic alterations (Crandall et al., 2011) involved in apoptosis (Crandall et al., 2011; Garcez et al., 2016) and cell cycle regulations (Crandall et al., 2011). The disruption of the normal retinoic acid signaling in developing forebrain has been shown to induce death of neural precursor cells and attenuate their growth with somewhat similar mechanisms (Rajaii et al., 2008), raising a possibility that ZIKV might be disrupting retinoic acid signaling pathway. Our hypothesis, hence derives support from known literature with multiple justifications. It explains many mysteries and helps in understanding the embryological basis of ZIKV mediated microcephaly.

Additionally, the white matter hypodensity noted in ZIKV mediated microcephaly (Hazin et al., 2016) can also be explained by dysregulation of retinoic acid signaling due to its noted involvement in myelination in the developing brain. To augment this, adequate retinoid receptor expression on oligodendrocyte precursor cells is necessary which later myelinate CNS (Huang et al., 2011). Dysregulation of retinoic acid signaling has also been implicated in some demyelinating pathologies (Okuda and Prados, 2009; König et al., 2012). Also, there is sufficient evidence that the retinal or other ophthalmic lesions, a unique finding observed in some ZIKV infected fetuses (de Paula Freitas et al., 2016; Ventura et al., 2016) may precipitate through disruption retinoic acid signaling (Mic et al., 2004; Micucci et al., 2014).

Our claim that ZIKV-microcephaly may involve retinoic acid signaling dysregulation is further bolstered by the fact that many of the toxins that involve retinoic acid also have microcephaly as a presenting feature (Lammer et al., 1985; Mochida and Walsh, 2001; Kot-Leibovich and Fainsod, 2009; Paganelli et al., 2010) and may share other malformations too. This is especially relevant to malformations arising in fetus with exposure of pregnant women to the isotretinoin (an analog of retinoic acid known to cause retinoic acid embryopathy). These malformations match to almost all the features described under ZIKV-microcephaly spectrum including enlarged cistern magna and calcifications in brain parenchyma (Lammer et al., 1985; Irving et al., 1986).

With the above-described pieces of evidence, a plausible mosaic of a justified picture comes up supporting our proposition that ZIKV interferes with retinoic acid signaling in the developing brain. In view of (1) the bioinformatic evidence of the presence of RARE sequence repeats in genome sequences of ZIKV strains and in other viruses known to cause congenital brain malformation (Table 3) and (2) confirmed role of retinoic acid in neural tube formation and further brain development mediated through RARE sequences, it is scientifically coherent to assume that a RARE sequence dependent mechanism could be the underlying mechanism of microcephaly seen in ZIKV infected fetuses. We, however stress that there is a need of confirming the proposed hypothesis on animal models or neural cell cultures.

Why embryonic defects are limited to the central nervous system only, and no anomaly of the other organs or limbs are reported in ZIKV if it causes an embryopathy is quite surprising. It could probably be due to strong neurotropic nature of the virus as it has been reported to be completely absent in organs other than the brain. A recent study performed the autopsy on ZIKV infected fetuses with severe microcephaly confirmed the absence of this virus in organs other than brain (Mlakar et al., 2016). ZIKV infection has also been observed to be less prevalent in human precursor cells other than the neural precursors (Crandall et al., 2011). Again, why did USG studies in ZIKV infected fetuses show no abnormality of brain structures almost until 20th weeks of gestation is a mystery and needs to be explained. A probable explanation for the late involvement of the brain structures has been indicated in the recent study based on immune-histological features of affected brain tissue. It indicates that time is consumed in virus invasion of the neurones and accumulation of approximate lesions capable of arresting cortical growth (Mlakar et al., 2016).

We chose retinoic acid for its probable role in ZIKV-mediated microcephaly but there is obvious need of exploring other molecular factors which may be involved in neural tube axis determination and its further development. Also, retinoic acid signaling dysregulation is not able to explain multifocal calcifications widespread in the basal ganglia, thalamus and cortical regions as noted in histological and radiological studies of ZIKV-microcephaly cases (Aragao et al., 2016; Calvet et al., 2016; Hazin et al., 2016; Mlakar et al., 2016). It is, however, important to mention that a rare presentation of an embryological anomaly syndrome (“CHARGE” syndrome, in which retinoic acid signaling dysregulation; Micucci et al., 2014) has been noted and reported with bilateral basal ganglia calcifications (Jain et al., 2011). Calcifications in brain parenchyma have also been mentioned in retinoic acid embryopathy (Lammer et al., 1985). As the calcifications found in the ZIKV affected fetal brain are punctiform and appear as destroyed neuronal structure on histological examination (Mlakar et al., 2016), an alternative explanation for their formation could be that calcifications are due to the deposition of the calcium compounds in the accumulations of viral invaded dead neurons (Mlakar et al., 2016) as the process of degeneration.

To test our hypothesis, it was is essential to ascertain that the ZIKV strains which contain either equal or less number of RARE sequences than Brazilian strain (Table 2) in their genome can also cause microcephaly or not. As we discussed above based the ZIKV strain which was recently implicated in microcephaly in Brazil has close similarity with the Polynesian and other two strains (Mlakar et al., 2016; Schuler-Faccini et al., 2016; Table 2, iv, viii, xiv), and all three contained the highest numbers of RARE consensus sequences in our study. But to make a valid claim to relate number of RARE consensus sequences with virulence of the strains, further availability of knowledge on the ZIKV strains implicated in the microcephaly is indispensable.

### Future prospects

The immediate application of our study are immense. If it is established that ZIKV caused microcephaly in infected fetus is a developmental condition arising out of retinoic acid signaling dysregulation it would not only help to understand the ZIKV–microcephaly pathogenesis but also may justify retinoic acid as a therapeutic target for preventing this condition.

## Author contributions

Conceived and designed the experiments: AK, HS, MF. Performed the experiments: AK, HS. Analyzed the data: AK, HS, MF. Wrote and revised the paper: AK, HS, VP, KR, SD, PK, SM, MF.

### Conflict of interest statement

The authors declare that the research was conducted in the absence of any commercial or financial relationships that could be construed as a potential conflict of interest.
